# First mtgenome sequences from three genera and phylogenetic relationships of the family Apidae based on mtgenome sequences (Hymenoptera: Apoidea)

**DOI:** 10.1080/23802359.2021.1903357

**Published:** 2021-03-23

**Authors:** Zhong-Quan Wen, Ting-Jing Li, Hua-Chuan Wang, Huan Yuan, Bin Chen

**Affiliations:** Chongqing Key Laboratory of Vector Insects, Institute of Entomology and Molecular Biology, Chongqing Normal University, Chongqing, China

**Keywords:** Apidae, mitochondrial genome, phylogenetics, *Thyreus decorus*, *Ceratina okinawana*, *Amegilla calceifera*

## Abstract

In this study, we report the complete mitochondrial genomes (mtgenome) of *Thyreus decorus*, *Ceratina okinawana* and *Amegilla calceifera*, which are the first time of mtgenome report also for the genera *Thyreus*, *Ceratina* and *Amegilla* in the family Apidae. They contain 15,237, 15,207, and 17,728 bp, with AT content of 84.97%, 79.30%, and 84.63%, respectively. Each mtgenome includes 13 protein-coding genes (PCGs), 22 transfer RNA (tRNAs), two ribosomal RNA (16S and 12S rRNA) and an AT-rich control region (CR). The phylogenetic relationships of 45 species in the family were constructed using Bayesian Inference based on concatenated nucleotide sequences of 13 PCGs. Our study suggests that the subfamily Apinae is a paraphyletic group, with the genus *Eucera* claded into the subfamily Xylocopinae and the genera *Amegilla* and *Thyreus* into the subfamily Nomadinae. In Apinae, the genera *Melipona* and *Bombus* are significantly sister group, and the genus *Apis* is the sister group with *Melipona* + *Bombus*.

The family Apidae has over known 5900 species, the largest family in the superfamily Apoidea in the number of species, is classified into three subfamilies (Apinae, Nomadinae, and Xylocopinae) and 210 genera (Ascher and Pickering 2020). Species of the family are considered to be the main pollinators of angiosperm, and have an indispensable influence in the normal operation of natural habitats and agricultural ecosystems (Williams 1994; Pywell et al. 2006; Ollerton et al. 2011). In addition, some species of the family play an important role in the economy and are used for the commercial production of honey and pollination of cultivated crops (Del Sarto et al. 2005). The phylogenetic relationships have largely unsettled in the family (Hedtke et al. 2013; Bossert et al. 2019). In this study, we sequenced and annotated the complete mtgenomes of *Thyreus decorus*, *Amegilla calceifera* and *Ceratina okinawana* also for the first time for their belonging genera in Apidae. We constructed and discussed the phylogenetic relationships of 45 known mtgenomes in Apidae.

The samples of *T. decorus*, *C. okinawana* and *A. calceifera* used for this study were collected from Guangxi (21°90.527 N, 107°90.365E), Guangxi (21°90.537 N, 107°90.448E) and Chongqing (28°90.881 N, 106°33.622E), China, respectively. Individual specimens were preserved in absolute ethanol after morphological identification and then stored at −80 °C before the DNA extraction. The total DNA was extracted from the muscle tissues using the DNeasy Blood and Tissue kit (250) (Qiagen 69506, Duesseldorf, Germany), and deposited in the Institute of Entomology and Molecular Biology, Chongqing Normal University, China (accession numbers: WZQ-TD-1, WZQ-CO-1 and WZQ-AC-1). The complete mitochondrial genomes of the three species were sequenced using the Illumina HiSeq system in the Shenzhen Huitong Biotechnology Limited Company (Shenzhen, China), and annotated using Mitos (http://mitos.bioinf.uni-leipzig.de/index.py) (Bernt et al. 2013). The PCGs and rRNAs genes were compared with the mtgenomes of related species, and the annotation results were confirmed using Geneious v4.8.5 (Kearse et al. 2012).

The complete mtgenomes of *T. decorus*, *C. okinawana* and *A. calceifera* are 15,237, 15,207 and 17,728 bp in length. They have been submitted to GenBank with accession numbers MW281318∼MH281320. Each mtgenome comprises of 37 typical mtgenome genes (13 PCGs, 22 tRNAs, and two rRNAs) and a control region (CR), consistent with other species in Apidae (Zhao et al. 2017). The mtgenomes of the three species have a high nucleotide bias with 84.97%, 79.30%, and 84.63% A + T content, and 15.03%, 20.70%, and 15.37% G + C content, respectively.

The phylogenetic relationships were constructed with the three newly sequenced and 42 known mtgenome sequences in Apidae, and *Megachile sculpturalis, Megachile strupigera* and *Osmia excavata* (Hymenoptera: Megachilidae) were used as outgroup. The 45 known species of mtgenome sequences were downloaded from GenBank. The Bayesian inference (BI) was employed for phylogenetic tree construction based on concatenated nucleotide sequences of 13 PCGs using PhyloSuite 1.2.1, and the best model was selected using ParririonFinder2 in the phylogenetic analysis (Ronquist et al. 2012; Zhang et al. 2020) (Figure 1). The result suggests that the subfamily Apinae is a paraphyletic group, with the genus *Eucera* clustered into the subfamily Xylocopinae and the genera *Amegilla* and *Thyreus* claded into the subfamily Nomadinae. In Apinae, the three genera *Melipona*, *Bombus* and *Apis* are obviously monophyletic with one of posterior probability for all of the three general clades. The genera *Melipona* and *Bombus* are sister group, and the genus *Apis* is the sister group with *Melipona* + *Bombus*. The genus *Eucera*, traditionally classified as Apinae, was grouped into Xylocopinae clade with 0.99 of posterior probability, and it maybe should be classified into the subfamily Xylocopinae, which is consistent with earlier studies (Zheng et al. 2018). Similarly, and the genera *Amegilla* and *Thyreue*, both traditionally classified to Apinae, were grouped into Nomadinae clade with 0.8 of posterior probability, and they maybe should be classified into the subfamily Nomadinae. Our results are basically consistent with the results of the earlier phylogenetic research results (Kawakita et al. 2008; Danforth et al. 2013). However, the genera *Eucera*, *Ceratina*, *Amegilla* and *Thyreue* have each only one mtgenome sequence to be included in the phylogenetic analysis; therefore, their phylogenetic positions need a further argument with more mtgenome sequences involved.

**Figure 1.**
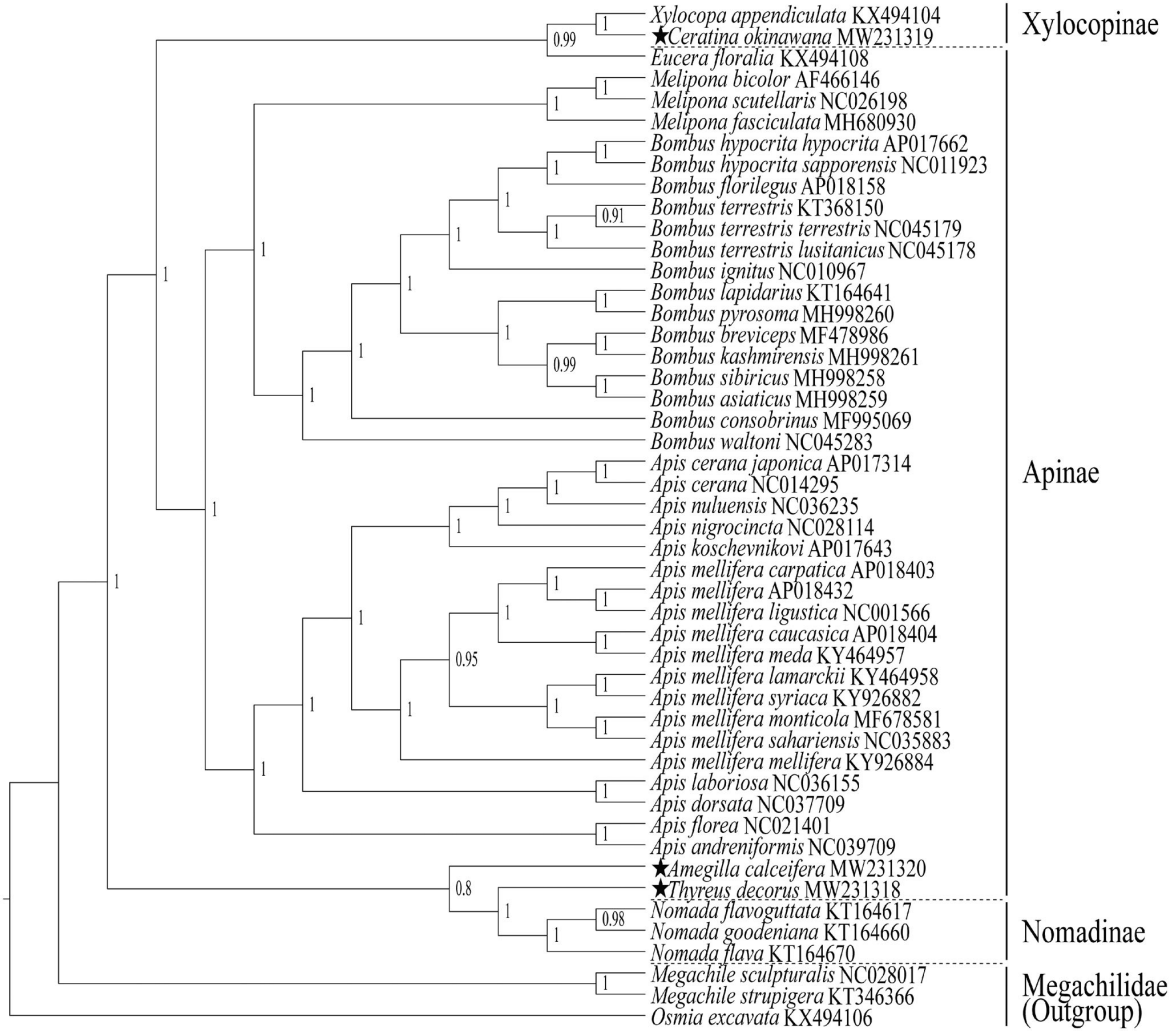
The phylogenetic relationships are constructed using Bayesian inference based on nucleotide sequences of 13 PCGs with 45 species of the family Apidae, and three species in the family Megachilidae are used as outgroup. The Bayesian posterior probabilities are showed at the nodes. The three newly sequenced mtgenomes are indicated by pentagrams.

## Data Availability

The genome sequence data that support the findings of this study are openly available in GenBank of NCBI at (https://www.ncbi.nlm.nih.gov/) under the accession no. MW281318-MW281320. The associated BioProject, SRA, and Bio-Sample numbers are PRJNA706124, PRJNA706149 and PRJNA706161, SRS8360571, SRS8361332 and SRS8361675, and SAMN18118499, SAMN18118864 and SAMN18119826 respectively.
